# Proposed new prognostic model using the systemic immune-inflammation index for primary central nervous system lymphoma: A prospective-retrospective multicohort analysis

**DOI:** 10.3389/fimmu.2022.1039862

**Published:** 2022-11-09

**Authors:** Shengjie Li, Zuguang Xia, Jiazhen Cao, Jinsen Zhang, Bobin Chen, Tong Chen, Xin Zhang, Wei Zhu, Danhui Li, Wei Hua, Ying Mao

**Affiliations:** ^1^ Department of Neurosurgery, Huashan Hospital, Fudan University, Shanghai, China; ^2^ National Center for Neurological Disorders, Shanghai, China; ^3^ Shanghai Key Laboratory of Brain Function Restoration and Neural Regeneration, Shanghai, China; ^4^ Neurosurgical Institute of Fudan University, Shanghai, China; ^5^ Shanghai Clinical Medical Center of Neurosurgery, Shanghai, China; ^6^ Department of Clinical Laboratory, Eye & ENT Hospital, Shanghai Medical College, Fudan University, Shanghai, China; ^7^ Department of Medical Oncology, Fudan University Shanghai Cancer Center, Fudan University, Shanghai, China; ^8^ Department of Oncology, Shanghai Medical College, Fudan University, Shanghai, China; ^9^ Department of Clinical Laboratory, Fudan University Shanghai Cancer Center, Shanghai, China; ^10^ Department of Hematology, Huashan Hospital, Fudan University, Shanghai, China; ^11^ Department of Pathology, RenJi Hospital, School of Medicine, Shanghai JiaoTong University, Shanghai, China

**Keywords:** Systemic immune-inflammation index, MSKCC, primary central nervous system lymphoma, prognosis, biomarker

## Abstract

**Purpose:**

The systemic immune-inflammation index (SII) has been considered a novel prognostic biomarker in several types of lymphoma. Our aims were to determine the best statistical relationship between pretreatment SII and survival and to combination of SII and the Memorial Sloan Kettering Cancer Center model (MSKCC) to derive the best prognostic mode in primary central nervous system lymphoma (PCNSL).

**Methods:**

Pretreatment SII and clinical data in 174 newly diagnosed PCNSL patients were included from two retrospective discovery cohorts (n = 128) and one prospective validation cohort (n = 46). A generalized additive model, Kaplan-Meier curve, and Cox analysis were performed. The high risk versus low risk of SII-MSKCC for the PCNSL cutoff point (0–1 vs. 2–4) was determined by the minimum P-value approach.

**Results:**

The SII showed a U-shaped relationship with the risk of overall survival (OS; P = 0.006). The patients with low SII or high SII had poorer OS and progression-free survival (PFS) than those with median SII. For PFS and OS, SII-MSKCC was a better predictor than MSKCC alone. The area under the receiver operating characteristic curve of the SII-MSKCC score was 0.84 for OS and 0.78 for PFS in the discovery cohorts. The predictive value of the SII-MSKCC score (OS, 0.88; PFS, 0.95) was verified through the validation cohort. Multivariable Cox analysis and Kaplan-Meier curve showed excellent performance for SII-MSKCC, with significant separation of two groups and better performance than MSKCC alone.

**Conclusions:**

We propose a new prognostic model using SII, age, and Karnofsky score that outperforms MSKCC alone and enables individualized estimates of patient outcome.

## Introduction

Primary central nervous system lymphoma (PCNSL) is a notorious extranodal non-Hodgkin lymphoma that is a heterogeneous and aggressive neoplasm. It has a high relapse rate and poor outcome (1). Currently, risk stratification and prognosis stratification of PCNSL is predominantly based on the clinical status according to the Memorial Sloan Kettering Cancer Center model (MSKCC) score including two variables (age and Karnofsky Performance Status score [KPS]), which was introduced in 2006 (2). Since then, developments in diagnosis and therapy have improved the prognosis of PCNSL. Therefore, although the MSKCC score remains prognostic, its ability to estimate survival has decreased (3, 4), which also casts doubt on the reliability of this two-parameter predictive model. The integration of other indices that are independent of traditional clinicopathologic indices into prognostic models could better characterize high-risk patients.

The systemic immune-inflammation index (SII) is defined as neutrophil count × platelet count/lymphocyte count, which indicates the immune and inflammatory state of the whole body. Recently, SII has been shown to be associated with the prognosis of malignant tumors (5–7). Although the precise mechanism remains unclear, previous studies have suggested that platelets could induce epithelial-mesenchymal transition in circulating tumor cells and promote tumor cell extravasation (8, 9). In the meantime, neutrophils can accelerate tumor adhesion and seeding of distant organ sites by secreting circulating growth factors (10, 11). Lymphocytes play a crucial role in tumor defense by inducing cytotoxic cell death (12). Therefore, SII has been reported to predict outcomes in patients with PCNSL (13, 14) and diffuse large b-cell lymphoma (DLBCL; 15–17). Currently, it is unknown how best to use SII, either alone or in combination with MSKCC factors, to estimate prognosis in PCNSL. We performed a multicenter cohort study including retrospective discovery and prospective validation cohorts to test the hypothesis that the combination of SII and MSKCC score (SII-MSKCC) could better characterize high-risk patients than MSKCC score alone.

The aims of this study were (1) to determine the best expression of the relationship between pretreatment SII and survival, (2) to compare the combination of SII with MSKCC and MSKCC alone to decide the best model to predict survival, and (3) to validate the best-performing model.

## Subjects and methods

### Study design and setting

This study was a prospective-retrospective analysis of three patient cohorts treated at three centers: Huashan Hospital, Renji Hospital, and Shanghai Cancer Center (18). The independent retrospective discovery cohorts were recruited at Huashan Hospital (n = 60) from January 1, 2010, to December 31, 2017, and at Renji Hospital (n = 68) from January 1, 2010, to December 31, 2017. The independent prospective validation cohort was recruited at the Shanghai Cancer Center (n = 46) from January 1, 2016, to December 31, 2020. Informed consent was obtained from all participants. The study protocol was approved by the institutional review board/ethics committee of Huashan Hospital, and the study adhered to the principles of the Declaration of Helsinki.

Clinical and demographic information was obtained from the medical data platform of each treatment center. Patient data included the following demographic and clinical information: age, sex, height, weight, blood pressure, hypertension, diabetes mellitus, Eastern Cooperative Oncology Group (ECOG) performance, lactate dehydrogenase, cerebrospinal fluid protein level, KPS score, medical history, date of diagnosis, tumor subsite, and so on. Classification of germinal center B cell-like (GCB) lymphoma and non-GCB lymphoma was determined using the Hans algorithm (19).

### Participants and management

Diagnosis of PCNSL was defined according to 2016 World Health Organization classification (20). All patients underwent a biopsy or a tumor resection, and positive CD-20 staining confirmed DLBCL (20). Patients could be eligible if they had magnetic resonance imaging (MRI) evidence of a brain parenchymal lesion showing homogeneous contrast enhancement indicative of lymphoma. All patients underwent a 2-deoxy-2[F-18] fluoro-D-glucose positron emission tomography/computed tomography (FDG PET/CT) scan and bone marrow aspiration to exclude systemic tumor manifestation based on the guidelines of the International PCNSL Collaborative Group (21) and the European Association of Neuro-Oncology (22). In brief, the inclusion criteria were age >18 years, histologic confirmation of PCNSL, negative human immunodeficiency virus serology, negative Epstein-Barr virus DNA, FDG PET/CT scan, and bone marrow biopsy.

The exclusion criteria were systemic lymphoma, leukemia, severe cardiac dysfunction, heart failure, symptomatic coronary artery disease, presence of third-space fluid such as pleural effusion or ascites, previous radiotherapy, previous chemotherapy, uncontrolled infection, multiple system organ failure, immunocompromised status, hepatitis B virus infection, hepatitis C virus infection, and positive pregnancy test.

All patients were treated with methotrexate-based combination immunochemotherapy. The detailed therapeutic schedule was the same as those previously described (23).

All patients were followed up at outpatient visits every three months during the first two years and every six months thereafter until death to remain up to date on patient survival status, disease progress, and time of death. Patients were followed up clinically and with MRI after therapy. If recurrence was suspected from clinical signs or symptoms, an MRI scan was done immediately. After progressive disease, patients were followed every three months for survival and returned to the previous follow-up schedule in the case of second remission. Laboratory monitoring, consisting of hematology, liver and renal function, and electrolytes, was done at baseline and before and after each administration of methotrexate.

### Analysis of blood sample

Laboratory tests were performed at the clinical laboratory department within each center. Peripheral blood samples were collected before the treatment. Blood samples were obtained in the morning through standard venipuncture from the antecubital fossa (anterior elbow vein) after the participants had fasted for 8 hours. Subsequently, 2-mL blood samples were collected in ethylenediaminetetraacetic acid tubes. Laboratory parameters were measured within 30 minutes after blood collection. Quantification of inflammatory and immunological cells was performed using an XN-Series automated blood counting system (Sysmex Co., Kobe, Japan). The SII was defined as neutrophil count × platelet count/lymphocyte count, so the unit of SII was 10^9^. The platelet-to-lymphocyte ratio (PLR) was defined as platelet count/lymphocyte count. The neutrophil-to-lymphocyte ratio (NLR) was defined as neutrophil count/lymphocyte count. The lymphocyte-to-monocyte ratio (LMR) was defined as lymphocyte count/monocyte count. Internal controls were analyzed daily for 10 years, with typical monthly coefficients of variation of 4% to 7% (platelets), 6% to 8% (monocytes), 2% to 5% (neutrophils), and 2% to 5% (lymphocytes). No significant changes were found in the coefficients of variation. The blood sample collection and detection method protocol was applied uniformly in both the retrospective cohort and the prospective cohort.

### Statistical analysis

We used an open-source calculator to calculate the minimum required sample size for diagnostic study based on specificity of 0.8 (allowable error, 0.1), sensitivity of 0.8 (allowable error, 0.1), α of 0.05 (2-tailed), and an overall incidence rate of 0.4 per 100 000 people. The sample size of each group was 44 so the sample size of each cohort can meet the requirements.

The primary endpoint was five-year overall survival (OS), defined as the time between diagnosis and death from any cause. Progression-free survival (PFS) was defined as the time between diagnosis and the first event, defined as death due to any cause or disease progression.

Normality was assessed using the Shapiro-Wilk test. The independent Student *t*-test, Kruskal-Wallis test, one-way analysis of variance, and χ^2^ test were used when appropriate. Baseline characteristics were expressed as frequency (percentage) or median (interquartile range [IQR]).

A generalized additive model (GAM) (24) with locally estimated scatterplot smoothing (LOESS) was used to identify the relationship between pretreatment SII levels and the risk of survival outcomes (detailed in the **Supplementary Material, Method**).

Receiver operating characteristic curve (ROC) analysis was performed. The adjusted.ROC function in the ROCt package to adjust for length of follow-up and age was used to produce the area under the receiver operating characteristic curve (AUC) value of SII and SII-MSKCC. The Youden index maximizing sensitivity plus specificity was applied to determine the best cutoff value for SII. SII was combined with MSKCC scores to establish a comprehensive model (SII [890 > SII > 450, score = 0; SII <450, score = 1; SII >890, score = 2]; MSKCC [age ≦50, score = 0; age >50 + KPS ≧70, score = 1; age >50 + KPS ≦70, score = 2). The high risk versus low risk for the PCNSL cutoff point was determined by a minimum P-value approach. We used SII-MSKCC (0–1 vs. 2–4) as a cutoff to investigate the study population.

The Kaplan-Meier method was used to estimate OS and PFS, and the log-rank test to assess the differences between the constructed plots. Univariate and multivariate Cox proportional hazards regression models were used to evaluate the association between SII levels and SII-MSKCC with clinical outcome (detailed in the Supplementary Material, Method). All analyses were performed using R statistical software (R-4.1.1, http://www.r-project.org) and SPSS (version 19.0; IBM Corp., Armonk, NY).

## Results

### Patient characteristics and even SII distribution in the three cohorts

**Figure 1:**
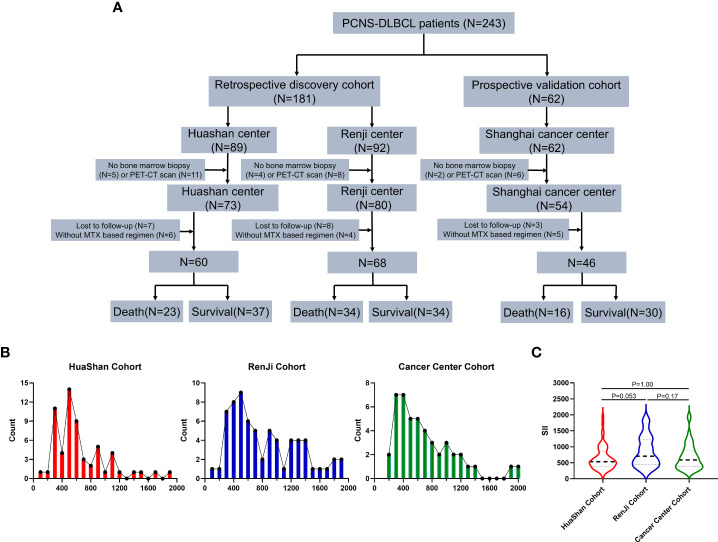
Study consort diagram and cohort description. **(A)** Study consort diagram: flowchart of the primary diffuse large B-cell lymphoma of the central nervous system study cohort, Discovery cohort 1: Huashan Center, Discovery cohort 2: RenJi Center, Validation cohort: Shanghai Cancer Center. **(B)** Distribution of pretreatment Systemic Immune-Inflammation Index (SII) in Huashan Center (Red), RenJi Center (Blu), and Shanghai Cancer Center (Green). **(C)** Pretreatment means SII levels across three centers. P values are derived from the Kruskall-Wallis test.

A total of 174 patients with newly diagnosed PCNSL were enrolled (**Figure 1A**). The detailed clinical characteristics of the three cohorts are summarized in **Table 1**. The median follow-up period was 35 months (IQR, 18–41 months), and the follow-up was unavailable in 9% (18/192) of patients. In the discovery cohort, 57 (45%) patients died and 77 (60%) experienced tumor recurrence. The median follow-up period was 36 months (IQR, 22–40 months). In the validation cohort, the median follow-up period was 35 months (IQR, 19–40 months), as 16 (35%) patients died and 23 (50%) experienced tumor recurrence. There was no statistical difference in the demographic and clinical characteristics between the discovery cohort and validation cohort, as shown in **Supplementary Table 1** (P > 0.05).

**Table 1:** Baseline characteristics in the discovery and validation cohorts.

Variables	All, N (%)	Discovery cohort 1, N (%)	Discovery cohort 2, N (%)	Validation cohort, N (%)
Number, n	174	60	68	46
Median age (IQR), y	62 (55-69)	65 (59-73)	59 (53-66)	59 (54-71)
≤60	79 (45)	17 (28)	38 (56)	24 (52)
>60	95 (55)	43 (72)	30 (44)	22 (48)
Male	118 (68)	31 (52)	42 (62)	25 (54)
Hypertension	56 (32)	16 (27)	25 (37)	15 (33)
Diabetes mellitus	15 (9)	6 (10)	5 (7)	4 (9)
Median BMI (IQR), Kg/m^2^	23.03 (21.99-24.98)	23.36 (20.49-24.66)	23.84 (21.74-25.39)	23.39 (22.04-25.45)
Missing	16 (9)	1 (2)	13 (19)	2 (4)
<18.5	9 (5)	6 (10)	1 (2)	2 (4)
18.5-24.0	105 (60)	43 (72)	37 (54)	25 (54)
>24.0	44 (25)	10 (17)	17 (25)	17 (37)
IELSG score				
Missing	42 (24)	15 (25)	15 (22)	12 (26)
0-3	112 (64)	40 (67)	42 (62)	30 (65)
4-5	20 (12)	5 (8)	11 (16)	4 (9)
MSKCC score				
Missing	14 (8)	3 (5)	8 (12)	3 (7)
Age≦50 or Age>50+KPS≧70	92 (53)	36 (60)	33 (49)	23 (50)
Age>50+KPS<70	68 (39)	21 (35)	27 (40)	20 (44)
ECOG performance				
Missing	9 (5)	1 (2)	2 (3)	6 (13)
0-2	105 (60)	44 (73)	35 (52)	26 (57)
3-5	60 (35)	15 (25)	31 (46)	14 (30)
IPI score				
Missing	14 (8)	3 (5)	4 (6)	7 (15)
0-2	122 (70)	45 (75)	44 (65)	33 (72)
3-5	38 (22)	12 (20)	20 (29)	6 (13)
LDH				
Missing	7 (4)	3 (5)	4 (6)	0 (0)
Normal	126 (72)	47 (78)	44 (65)	35 (76)
Decreased	15 (9)	2 (3)	8 (12)	5 (11)
Elevated	26 (15)	8 (13)	12 (18)	6 (13)
Cell of origin				
Germinal	43 (25)	13 (22)	14 (21)	16 (35)
Non-germinal center	131 (75)	47 (78)	54 (79)	30 (65)
Treatment				
MTX	44 (25)	14 (23)	18 (26)	12 (26)
MTX-based regimen	130 (75)	46 (77)	50 (74)	34 (74)
Death				
Yes	73 (42)	23 (38)	34 (50)	16 (35)
No	101 (58)	37 (62)	34 (50)	30 (65)
Progression				
Yes	100 (57)	36 (60)	41 (60)	23 (50)
No	74 (43)	24 (40)	27 (40)	23 (50)

IQR, interquartile range; BMI, body mass index; LDH, lactic dehydrogenase.

The distributions of SII in the Huashan (P = 0.0010), Renji (P = 0.0040), and Shanghai cancer centers (P < 0.0001) were non-normal. After a logarithmic transformation, the distributions of log SII in Huashan (P = 0.18), Renji (P = 0.096), and Shanghai (P = 0.60) were normal. The distribution of SII by center is shown in **Figure 1B**, and the distribution of SII was similar across the three cohorts. There was no significant difference in SII levels across the three centers (P > 0.05; **Figure 1C**).

### The determination of SII threshold and clinical characteristics in the discovery cohort

Overall, 101 patients (79%) with PCNSL were identified as having the non-GCB-derived subtype and 27 patients (21%) were identified as having the GCB subtype in the discovery cohort (**Supplementary Table 2**). Molecular classification (GCB vs. non-GCB) was not associated with OS (**Supplementary Figure 1A**) or PFS (**Supplementary Figure 1B**) in the Cox proportional hazards model. A higher International Prognostic Index (IPI) score (**Supplementary Figures 1C, D**) and a higher International Extranodal Lymphoma Study Group (IELSG) score (**Supplementary Figures 1E, F**) were associated with shorter OS and PFS.

In comparison with patients showing 450 < SII < 890, those with SII <450 or SII >890 shared worse ECOG (P < 0.05) and IPI performance (P < 0.0001) and worse outcomes (OS, P < 0.05; PFS, P < 0.05). Moreover, those with SII <450 (mean age, 65 years) or SII >890 (mean age, 65 years) were slightly older (P = 0.47) than those with 450 < SII < 890 (mean age, 59 years). A comparison of clinical characteristics between patients with pretreatment SII <450 or SII >890 and 450 < SII < 890 is detailed in **Supplementary Table 3**.

### Higher and lower SII could be associated with poor outcome in PCNSL

**Figure 2:**
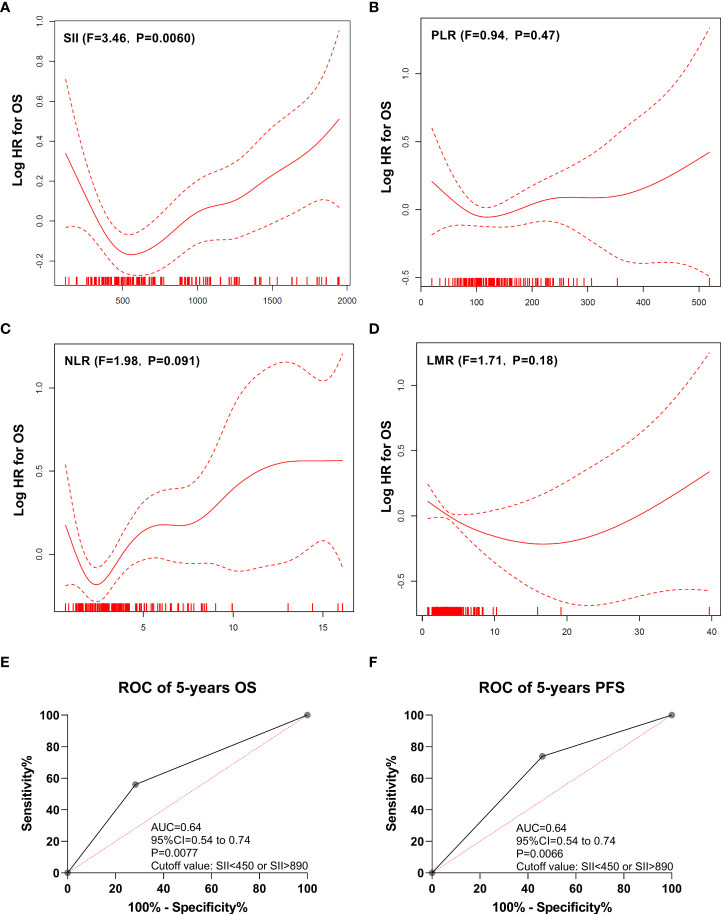
Generalized additive model plot and ROC curve. **(A)** Generalized additive model plot: in the discovery cohort, a U-shape correlation between pretreatment systemic immune-inflammation index (SII) and risk of overall survival. **(B)** Generalized additive model plot: in the discovery cohort, no linear or nonlinear relationship between platelet to lymphocyte ratio (PLR) and the risk of overall survival. **(C)** Generalized additive model plot: in the discovery cohort, no linear or nonlinear relationship between neutrophil to lymphocyte ratio (NLR) and the risk of overall survival. **(D)** Generalized additive model plot: in the discovery cohort, no linear or nonlinear relationship between lymphocyte to monocyte ratio (LMR) and the risk of overall survival. **(E)** ROC curve of overall survival. **(F)** ROC curve of progression-free survival. The generalized additive model plot was expressed as the logarithm of the odds (logit) **(A-D)**. The solid line shows the fitted values using a generalized additive model and the dotted curve shows the upper and lower 95% confidence intervals **(A-D)**.

The GAM plot revealed a U-shaped relationship between SII (F = 3.46, P = 0.0060; **Figure 2A**) and OS in the discovery cohort. However, PLR (F = 0.94, P = 0.47; **Figure 2B**), NLR (F = 1.98, P = 0.091; **Figure 2C**), and LMR (F = 1.71, P = 0.18; **Figure 2D**) showed no linear or nonlinear relationship with OS. The ROC analysis was used to define the cutoff of SII to distinguish prognosis, and the best cutoff values of SII were SII <450 or SII >890, with an AUC of 0.64 (95% confidence interval [CI]: 0.54–0.74, P = 0.0077), as shown in **Figure 2E**. In addition, SII <450 or SII >890 also could distinguish PFS, and the AUC was 0.64 (**Figure 2F**).

**Figure 3:**
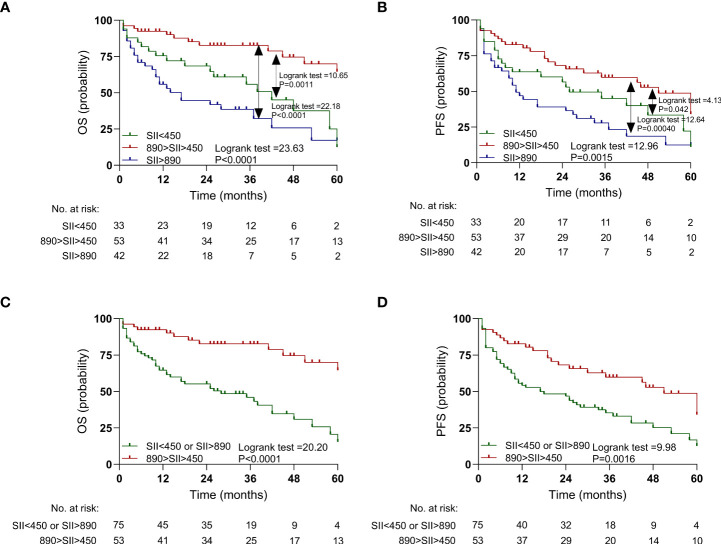
Kaplan-Meier estimates overall survival (OS) and progression-free survival (PFS) according to pretreatment systemic immune-inflammation index (SII) levels in the discovery cohort. **(A)** OS according to SII<450, 450<SII<890, and SII>890 three subgroups. **(B)** PFS according to SII<450, 450<SII<890, and SII>890 three subgroups. **(C)** OS according to SII<450 or SII>890, and 450<SII<890 two subgroups. **(D)** PFS according to SII<450 or SII>890, and 450<SII<890 two subgroups.

The impact of the SII on PFS and OS was analyzed, and the patients with SII >890 had a poorer OS (P < 0.0001; **Figure 3A**) and PFS (P = 0.00040; **Figure 3B**) than those with a median SII value (890 > SII > 450). Similarly, the patients with SII <450 had a poorer OS (P = 0.0011; **Figure 3A**) and PFS (P = 0.042; **Figure 3B**) than those with a median SII value (890 > SII > 450). The Huashan (**Supplementary Figures 2A, B**) and Renji (**Supplementary Figures 2C, D**) cohorts showed similar results.

Furthermore, SII <450 and SII >890 were combined into one group. The patients with SII <450 or SII >890 had a worse OS (P < 0.0001; **Figure 3C**) and PFS (P = 0.0016; **Figure 3D**) than did those with median SII values (450 < SII < 890). Similar results were also observed in the Huashan (**Supplementary Figure 3A**) and Renji cohorts (**Supplementary Figures 3C, D**).

Cox regression analysis was performed to evaluate the influence of SII as a predictor of clinical outcomes in the context of contemporary prognostic factors in the discovery cohort (**Supplementary Table 4**). In a multivariable model that included all known contemporary prognostic features, SII <450 or SII >890 levels retained prognostic significance for OS (hazard ratio [HR]: 3.80, P = 0.0004) and PFS (HR: 3.43, P = 0.0020). Similar results were observed in the Renji (**Supplementary Table 5**) and Huashan centers (**Supplementary Table 6**).

### The prognostic value of SII could be validated in an independent cohort

**Figure 4:**
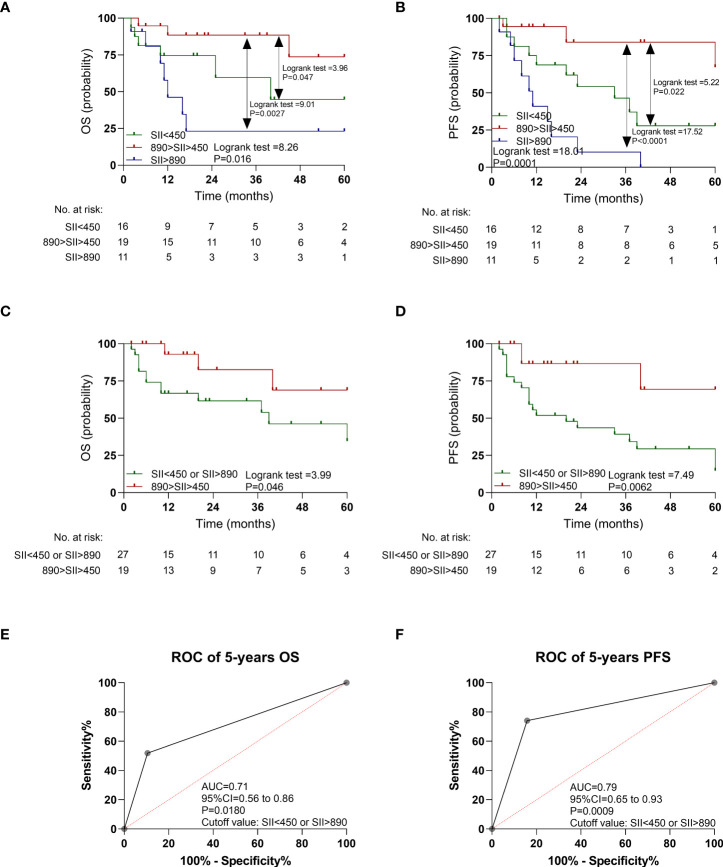
Kaplan-Meier estimates overall survival (OS) and progression-free survival (PFS) according to pretreatment systemic immune-inflammation index (SII) levels, and ROC curve in the validation cohort. **(A)** OS according to SII<450, 450<SII<890, and SII>890 three subgroups. **(B)** PFS according to SII<450, 450<SII<890, and SII>890 three subgroups. **(C)** OS according to SII<450 or SII>890, and 450<SII<890 two subgroups. **(D)** PFS according to SII<450 or SII>890, and 450<SII<890 two subgroups. **(E)** ROC curve of OS. **(F)** ROC curve of PFS.

In the validation cohort, the patients with SII <450 had a poorer OS (P = 0.047; **Figure 4A**) and PFS (P = 0.022; **Figure 4B**) than those with median SII (450 < SII < 890), as shown in **Figure 4** and **Supplementary Table 6**. Similarly, the patients with SII >890 had a poorer OS (P = 0.0027; **Figure 4A**) and PFS (P < 0.0001; **Figure 4B**) than did those with median SII (450 < SII < 890). In addition, the patients with SII >890 or SII <450 together also had a poorer OS (P = 0.046; **Figure 4C**) and PFS (P = 0.0062; **Figure 4D**) than those with median SII (450 < SII < 890).

As shown in **Figures 4E, F**, the AUC of SII <450 or SII >890 to distinguish OS and PFS was 0.71 (95% CI: 0.56–0.86, P = 0.0180) and 0.79 (95% CI: 0.65–0.93, P = 0.0009). The multivariable Cox regression analysis indicated that SII <450 or SII >890 still retained prognostic significance for OS (HR: 3.97, P = 0.0034) and PFS (HR: 8.23, P = 0.0010), respectively (**Table 2**).

**Table 2:** Univariable analysis and multivariable analysis for progression-free survival and overall survival in validation cohort.

	OS		PFS	
	HR (95%CI)	P value	HR (95%CI)	P value
Univariate analysis				
Model 1				
890>SII>450	1		1	
SII<450	3.20 (0.79-12.97)	0.10	5.48 (1.49-20.11)	0.010
SII>890	6.23 (1.59-24.43)	0.0093	15.67 (3.37-72.95)	<0.0001
Model 2				
SII				
890>SII>450	1		1	
SII<450 or SII>890	4.25 (1.20-15.01)	0.025	7.47 (2.19-25.42)	0.0012
MSKCC				
Age≦50 or Age>50+KPS≧70	1		1	
Age>50+KPS<70	11. 75 (1.37-30.81)	0.025	6.91 (2.52-15.97)	<0.001
Model 3				
SII-MSKCC (0-1)	1		1	
SII-MSKCC (2-4)	26.75 (3.46-66.67)	0.0020	41.33 (5.45-113.62)	<0.0001
Multivariate analysis				
Model 1*				
890>SII>450	1		1	
SII<450	5.24 (1.08-14.14)	0.040	5.64 (1.52-10.94)	0.010
SII>890	8.50 (1.70-22.60)	0.0090	16.92 (3.51-41.82)	<0.0001
Model 2*				
SII				
890>SII>450	1		1	
SII<450 or SII>890	3.97 (1.11-8.18)	0.034	8.23 (2.36-18.69)	0.0010
MSKCC				
Age≦50 or Age>50+KPS≧70	1		1	
Age>50+KPS<70	13.19 (2.97-36.88)	0.033	29.41 (5.12-79.13)	<0.001
Model 3**				
SII-MSKCC (0-1)	1		1	
SII-MSKCC (2-4)	43.07 (3.04-11.044)	0.0050	90.03 (5.74-280.66)	0.0020

*Adjusted for sex (male = 1, female = 2), BMI, MSKCC, diabetes (yes = 1, no = 0), and hypertension (yes = 1, no = 0). **Adjusted for age, sex (male = 1, female = 2), BMI, diabetes (yes = 1, no = 0), and hypertension (yes = 1, no = 0). A total of 42 patients (death=16, progression=21) without missing values included in this multivariable analysis model.

### The prognostic value of MSKCC score

As shown in **Supplementary Figure 4**, the AUC of MSKCC to distinguish OS (**Supplementary Figure 4A**) and PFS (**Supplementary Figure 4B**) was 0.75 and 0.78 in the discovery cohort. In the validation cohort, the AUC of MSKCC to distinguish OS (**Supplementary Figure 4C**) and PFS (**Supplementary Figure 4D**) was 0.75 and 0.82 in the discovery cohort.

Additionally, a higher MSKCC score was also associated with shorter OS (HR:10.22, 95% CI: 5.46–19.12, P < 0.0001; **Supplementary Figure 1G**) and PFS (HR: 4.87, 95% CI: 2.86–8.29, P < 0.0001; **Supplementary Figure 1H**).

### SII-MSKCC scores could be established with a comprehensive model

In the discovery cohort, the patients with higher SII-MSKCC scores had a poorer OS (P < 0.0001; **Supplementary Figure 5A**) and PFS (P < 0.0001; **Supplementary Figure 5B**) than those with lower SII-MSKCC scores. Similarly, the patients with higher SII-MSKCC scores had a poorer OS (P < 0.0001; **Supplementary Figure 5C**) and PFS (P < 0.0001; **Supplementary Figure 5D**) than those with lower SII-MSKCC scores in the validation cohort.

**Figure 5:**
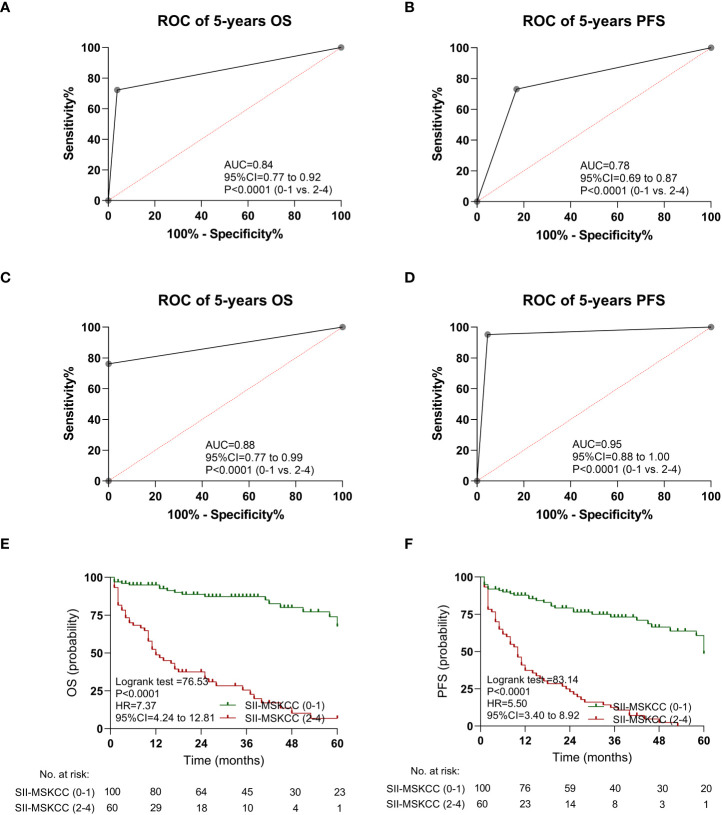
Kaplan-Meier estimates overall survival (OS) and progression-free survival (PFS) according to SII-MSKCC scores, and the ROC curve of SII-MSKCC. **(A)** ROC curve of OS in the discovery cohort. **(B)** ROC curve of PFS in the discovery cohort. **(C)** ROC curve of OS in the validation cohort. **(D)** ROC curve of PFS in the validation cohort. **(E)** OS according to SII-MSKCC model scores (0-1 *vs.* 2-4). **(F)** PFS according to SII-MSKCC scores (0-1 *vs.* 2-4).

In the discovery cohort, the AUC value of SII-MSKCC was 0.84 (95% CI: 0.77–0.92, P < 0.0001) of OS (**Figure 5A**) and 0.78 (95% CI: 0.69–0.87, P < 0.0001) of PFS (**Figure 5B**). Moreover, the predictive value (OS, AUC = 0.88, **Figure 5C**; PFS, AUC = 0.95, **Figure 5D**) of the SII-MSKCC model was verified through the clinical prospective cohort we established.

### The SII-MSKCC score model could better predict prognosis in patients with PCNSL

In a multivariable model that included all known contemporary prognostic features, SII-MSKCC scores retained prognostic significance for OS (HR: 6.44, 95% CI: 2.90–14.31, P < 0.0001) and PFS (HR: 3.24, 95% CI: 1.75–5.98, P < 0.0001) in the discovery cohort (**Supplementary Table 4**). Similar results were also observed in the validation cohort (**Table 2**).

The impact of the SII-MSKCC on PFS and OS was determined by Kaplan-Meier analysis, and the patients with high SII-MSKCC scores (2–4) had a shorter OS (HR: 7.37, 95% CI: 4.24–12.81, P < 0.0001; **Figure 5E**) and PFS (HR: 5.50, 95% CI: 3.40–8.92, P < 0.0001; **Figure 5F**) than those with low SII-MSKCC scores (0–1).

Furthermore, the patients with high SII-MSKCC scores (2–4) had a shorter three-year OS (HR: 8.75, 95% CI: 4.83–15.85, P < 0.0001; **Supplementary Figure 6A**) and three-year PFS (HR: 5.65, 95% CI: 3.41–9.37, P < 0.0001; **Supplementary Figure 6B**) than those with a low SII-MSKCC scores (0–1). Similar results were also observed in the validation cohort (OS, **Supplementary Figure 6C**; PFS, **Supplementary Figure 6D**).

## Discussion

We present a simple and robust prognostic model (SII-MSKCC) that predicts outcomes for PCNSL better than MSKCC alone. Our newly developed model is dependent on three identified variables: age, KPS score, and SII. In the discovery cohort, the AUC value of the SII-MSKCC model was 0.84 for OS and 0.78 for PFS, and this was verified through the clinical prospective cohort we established. Compared with single SII and MSKCC prognosis variables, the SII-MSKCC model could better identify a subgroup of patients with favorable long-term survival both in the discovery cohort and validation cohort.

Systemic immune-inflammation index was reported to be associated with OS in PCNSL (13, 14) and DLBCL (15–17) in a linear manner. However, a U-shaped relationship was first demonstrated here, which is different from previous studies (5, 25). Our results showed that the patients with low or high SII had worse outcomes than those with median SII in the discovery cohort. When analyzed with Cox regression, the HRs were significant in both univariate and multivariate models, suggesting low SII or high SII as an independent prognostic factor for PCNSL. Meanwhile, it was also demonstrated in the validation cohort. In terms of the finding for high SII, it was associated with worse survival, which is consistent with previous reports of DLBCL (16, 17) and PCNSL (13, 14). Interestingly, the patients with low SII also had worse outcomes, which has not been reported in other studies. The explanations would include differences in the cohort sample size (single cohort vs. multi cohort), sample type (PCNSL vs. DLBCL), and statistical methodology. The GAM plot analysis was necessary to explore the correlation between SII and clinical outcome, which was ignored in previous studies.

The biological reasons for the underlying strong U-shaped relationship between SII and prognosis remain to be elucidated. The reasons may be as follows: (1) The inflammatory microenvironment is an intrinsic feature of cancer, which could accelerate the aggregation and secretion of proinflammatory cytokines, angiogenic and lymphogenic factors, and oncogenic chemokines (12, 26, 27). Thus, SII >890 can be regarded as a biomarker for poor prognosis. (2) A low level of SII might indicate an immunosenescent status, and immunosenescence could alter the immune response, leading to tumor escape and progression (28–30). In this study, we also found that those with SII <450 (mean age, 65 years) were slightly older than those with 450 < SII < 890 (mean age, 59 years). So, SII <450 can also be considered as a surrogate biomarker for poor outcome. More research on this topic needs to be undertaken.

We confirmed that high IPI, IELSG, and MSKCC scores were associated with worse prognosis in patients with PCNS-DLBCL, which was consistent with previous studies (31–33). Meanwhile, we found that those with SII >890 or SII <450 had a poorer IPI performance in comparison with patients showing 890 > SII > 450. In a multivariable Cox regression analysis model adjusted for MSKCC score and all other known contemporary prognostic features, SII was also a marker that discriminated groups of patients with different survival. A larger cohort study is required to confirm these results.

In this study, in comparison with patients showing 450 < SII < 890, those with SII <450 or SII >890 had worse ECOG and IPI performance. Wang et al. (15) reported that DLBCL patients with high SII tended to have poor ECOG performance and high IPI score. Liu et al. (34) also reported that interleukin-2 and tumor necrosis factor alpha were positively correlated with ECOG score in multiple myeloma. Our results showed that high SII was associated with aggressive clinical features, which is consistent with previous studies, indicating that poor ECOG and high IPI scores were associated with a high inflammatory status. Limited data could be available in the literature regarding the association between low SII and ECOG performance and IPI scores. A low SII indicated an immunosenescent status. Meanwhile, immunosenescence can lead to tumor escape and progression (28). We speculate that the patients with poor ECOG performance and high IPI score may have a high degree of immunosenescence. Thus, poor ECOG performance and high IPI scores were associated with a low SII level. The results warrant further validation in larger prospective cohorts.

Our study has multiple strengths. First, to our knowledge, this is the first prospective report highlighting the prognostic validity of SII in PCNSL, and a U-shape—not a linear correlation—was observed here. Second, this study is the largest prospective-retrospective designed multicenter study of patients with PCNSL. Furthermore, a novel prognostic score, the SII-MSKCC score, was established, which may be highly recommended as a novel predictive tool to greatly improve the accuracy of identifying a subgroup of patients with favorable long-term survival.

Our study also had several limitations. First, it did not include genomic markers in risk group classification. Second, this was a prospective-retrospective multicenter study, and data collection and methods may have differed across the centers in the retrospective discovery cohorts. Therefore, some potential clinical variables that may affect outcomes were not available for all patients. Last, overall performance status will vary in patients with central nervous system involvement, but in addition, various other factors like site of involvement (e.g., brainstem vs. cortical), presence of hydrocephalus, amount of perilesional edema, and other features of impending herniation may have far more prognostic relevance. However, because this was a prospective-retrospective multicenter study, these factors were not included in this study. Therefore, these clinical variables may affect outcomes.

In conclusion, we present a simple, robust new prognostic model that can be used in clinical practice and clinical trials for adults with newly diagnosed PCNSL. By combining pretreatment SII, age, and KPS score, it allows individualized estimates of patient outcome.

## Data availability statement

The raw data supporting the conclusions of this article will be made available by the authors, without undue reservation.

## Ethics statement

The studies involving human participants were reviewed and approved by the institutional review board/ethics committee of Huashan Hospital. The patients/participants provided their written informed consent to participate in this study. Written informed consent was obtained from the individual(s) for the publication of any potentially identifiable images or data included in this article.

## Author contributions

SL: Data curation, formal analysis, investigation, visualization, writing–original draft, project administration, writing–review and editing. ZX: Data curation, formal analysis, investigation, visualization, writing–original draft, writing–review and editing. DL: Data curation, formal analysis, investigation, visualization, writing–original draft, project administration, writing–review and editing. WH: Resources, supervision, funding acquisition, investigation, writing–original draft, writing–review and editing. YM: Resources, supervision, funding acquisition, investigation, writing–original draft, writing–review and editing. JC: Formal analysis, investigation, methodology, writing–original draft, writing–review and editing. JZ: Formal analysis, visualization, methodology, writing–original draft, writing–review and editing. XZ: Formal analysis, visualization, methodology, writing–original draft, writing–review and editing. BC: Data curation, formal analysis, investigation, writing–original draft, writing–review and editing. TC: Formal analysis, visualization, methodology, writing–original draft, writing–review and editing. WZ: Formal analysis, visualization, methodology, writing–original draft, writing–review and editing. All authors contributed to the article and approved the submitted version.

## Funding

The study was funded by Shanghai Science and Technology Commission (17430750200), Join Breakthrough Project for New Frontier Technologies of Shanghai Hospital Development Center (SHDC12016120), CAMS Innovation Fund for Medical Sciences (CIFMS, 2022-I2M-C&T-B-112), Youth Medical Talents – Clinical Laboratory Practitioner Program (2022-65).

## Acknowledgments

The authors would like to thank Genomicare Biotechnology (Shanghai) for their assistance in patient’s follow-up. The authors would like to thank Jianchen Fang (RenJi Hospital, Shanghai JiaoTong University) for his advice in the immunohistochemistry staining evaluation.

## Conflict of interest

The authors declare that the research was conducted in the absence of any commercial or financial relationships that could be construed as a potential conflict of interest.

## Publisher’s note

All claims expressed in this article are solely those of the authors and do not necessarily represent those of their affiliated organizations, or those of the publisher, the editors and the reviewers. Any product that may be evaluated in this article, or claim that may be made by its manufacturer, is not guaranteed or endorsed by the publisher.
