# Supplementary material for: Proposed new prognostic model using the systemic immune-inflammation index for primary central nervous system lymphoma: A prospective-retrospective multicohort analysis
Source: Front Immunol. 2022 Nov 9;13:1039862. doi: 10.3389/fimmu.2022.1039862 (PMC9681794; doi:10.3389/fimmu.2022.1039862)
Supplement: Supplementary file 1 [file DataSheet_1.pdf]

Proposed new prognostic model using the systemic immune-inflammation index for primary central nervous system lymphoma: A prospective-retrospective multicohort analysis

Shengjie Li[1,2,3,4,5,6#], Zuguang Xia[7,8#], Jiazhen Cao[8,9], Jinsen Zhang[1,2,3,4,5], Bobin Chen[10], Tong Chen[10], Xin Zhang1[,2,3,4,5], Wei Zhu[1,2,3,4,5], Danhui Li[11*], Wei Hua[1,2,3,4,5*], Ying Mao[1,2,3,4,5*]

1. Department of Neurosurgery, Huashan Hospital, Fudan University, Shanghai, 200040, China.

2. National Center for Neurological Disorders, Shanghai, 200040, China.

3. Shanghai Key Laboratory of Brain Function Restoration and Neural Regeneration, Shanghai, 200040, China.

4. Neurosurgical Institute of Fudan University, Shanghai, 200040, China.

5. Shanghai Clinical Medical Center of Neurosurgery, Shanghai, 200040, China.

6. Department of Clinical Laboratory, Eye & ENT Hospital, Shanghai Medical College, Fudan University, Shanghai, 200031, China.

7. Department of Medical Oncology, Fudan University Shanghai Cancer Center, Fudan University, Shanghai 200032, China.

8. Department of Oncology, Shanghai Medical College, Fudan University, Shanghai 200032, China.

9. Department of Clinical Laboratory, Fudan University Shanghai Cancer Center, Shanghai, 200032, China.

10. Department of Hematology, Huashan Hospital, Fudan University, Shanghai, 200040, China.

11. Department of Pathology, RenJi Hospital, School of Medicine, Shanghai JiaoTong University, Shanghai, 200127, China.

#Shengjie Li and Zuguang Xia contributed equally to this work.

Corresponding Author:

Danhui Li, Department of Pathology, RenJi Hospital, School of Medicine, Shanghai JiaoTong University, No. 160 PuJian Road, Shanghai, 200127, China; Electronic address: danhuili1002@163.com.

Wei Hua, Department of Neurosurgery, Huashan Hospital, Fudan University, No. 12 Wulumuqi Road, Shanghai, 200040, China; Electronic address: hs_huawei@126.com.

Ying Mao, Department of Neurosurgery, Huashan Hospital, Fudan University, No. 12 Wulumuqi Road, Shanghai, 200040, China; Electronic address: maoying@fudan.edu.cn.

Contents

(1) Supplementary Figure 1. Kaplan-Meier estimates of overall survival (OS) and progression-free survival (PFS) according to molecular classification, IPI scores, IELSG scores, and MSKCC scores in the discovery cohort

(2) Supplementary Figure 2. Kaplan-Meier estimates of overall survival (OS) and progression-free survival (PFS) according to pretreatment systemic immune-inflammation index (SII) levels in the discovery cohort 1 (HuaShan Center) and discovery cohort 2 (RenJi Center)

(3) Supplementary Figure 3. Kaplan-Meier estimates of overall survival (OS) and progression-free survival (PFS) according to pretreatment systemic immune-inflammation index (SII) levels in the discovery cohort 1 (HuaShan Center) and discovery cohort 2 (RenJi Center)

(4) Supplementary Figure 4. ROC curve of MSKCC

(5) Supplementary Figure 5. Kaplan-Meier estimates of overall survival (OS) and progression-free survival (PFS) according to SII-MSKCC scores

(6) Supplementary Figure 6. Kaplan-Meier estimates 3 years overall survival (OS) and 3 years progression-free survival (PFS) according to SII-MSKCC scores.

(7) Supplementary Table 1. Demographic and clinical characteristics between discovery cohort and validation cohort

(8) Supplementary Table 2. The results of immunohistochemistry staining

(9) Supplementary Table 3. Patient characteristics and outcome in discovery cohort by SII groups

(10) Supplementary Table 4. Univariable analysis and multivariable analysis for progression-free survival and overall survival in discovery cohort

(11) Supplementary Table 5. Univariable analysis and multivariable analysis for progression-free survival and overall survival in RenJi Cohort

(12) Supplementary Table 6. Univariable analysis and multivariable analysis for progression-free survival and overall survival in HuaShan Cohort

(13) Supplementary method

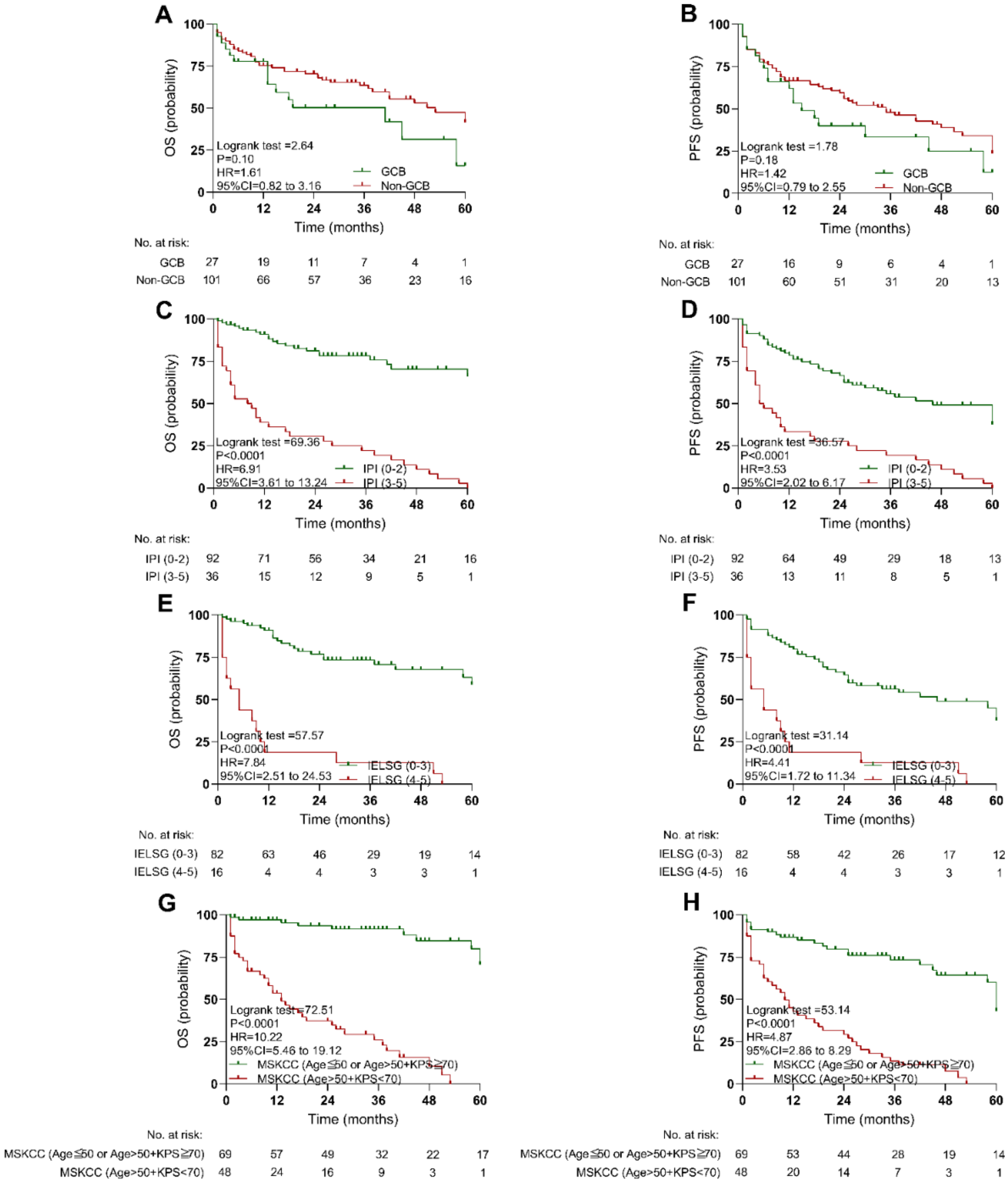

Supplementary Figure 1. Kaplan-Meier estimates of overall survival (OS) and progression-free survival (PFS) according to molecular classification, IPI scores, IELSG scores, and MSKCC scores in the discovery cohort. (A) OS according to molecular classification (GCB vs Non-GCB). (B) PFS according to molecular classification (GCB vs Non-GCB). (C) OS according to IPI scores (0-2 vs 3-5). (D) PFS according to IPI scores (0-2 vs 3-5). (E) OS according to IELSG scores (0-3 vs 4-5). (F) PFS according to IELSG scores (0-3 vs 4-5). (G) OS according to MSKCC scores. (H) PFS according to MSKCC scores.

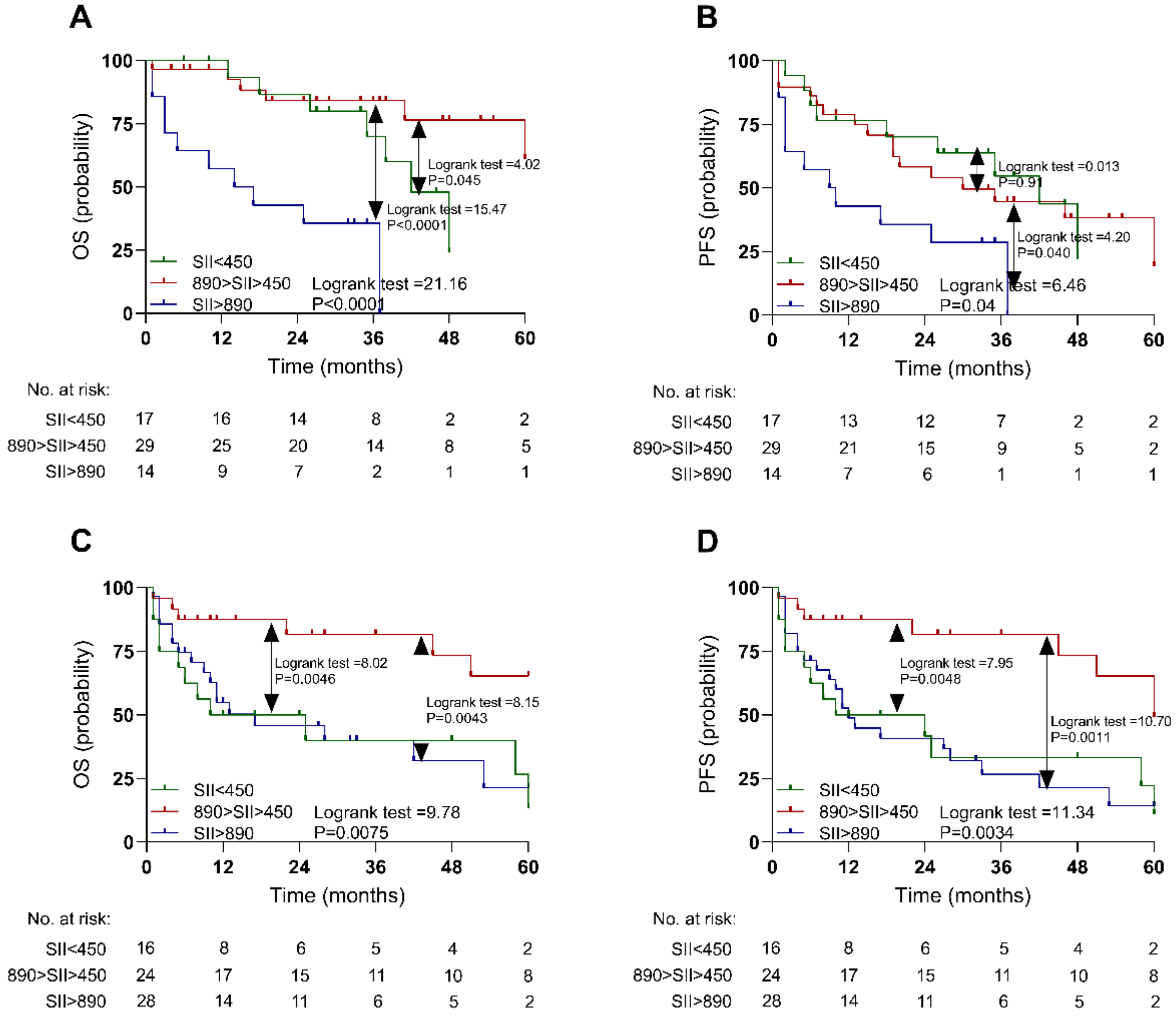

Supplementary Figure 2. Kaplan-Meier estimates of overall survival (OS) and progression-free survival (PFS) according to pretreatment systemic immune-inflammation index (SII) levels in the discovery cohort 1 (HuaShan Center) and discovery cohort 2 (RenJi Center). (A) OS according to SII<450, 890>SII>450, and SII>890 three subgroup in HuaShan Center. (B) PFS according to SII<450, 890>SII>450, and SII>890 three subgroup in HuaShan Center. (C) OS according to SII<450, 890>SII>450, and SII>890 three subgroup in RenJi Center. (D) PFS according to SII<450, 890>SII>450, and SII>890 three subgroup in RenJi Center.

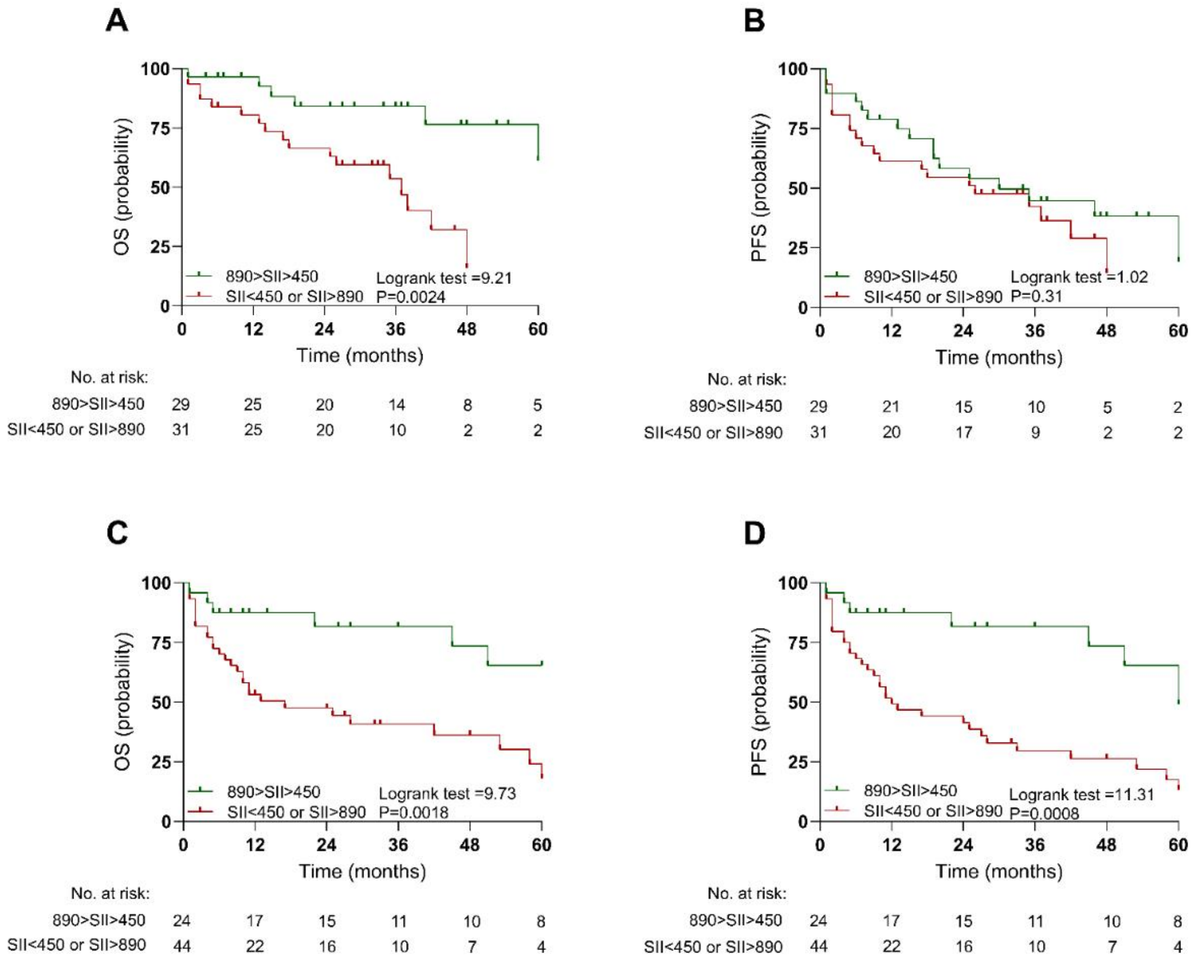

Supplementary Figure 3. Kaplan-Meier estimates of overall survival (OS) and progression-free survival (PFS) according to pretreatment systemic immune-inflammation index (SII) levels in the discovery cohort 1 (HuaShan Center) and discovery cohort 2 (RenJi Center). (A) OS according to SII<450 or SII>890, and 890>SII>450 two subgroup in HuaShan Center. (B) PFS according to SII<450 or SII>890, and 890>SII>450 two subgroup in HuaShan Center. (C) OS according to SII<450 or SII>890, and 890>SII>450 two subgroup in RenJi Center. (D) PFS according to SII<450 or SII>890, and 890>SII>450 two subgroup in RenJi Center.

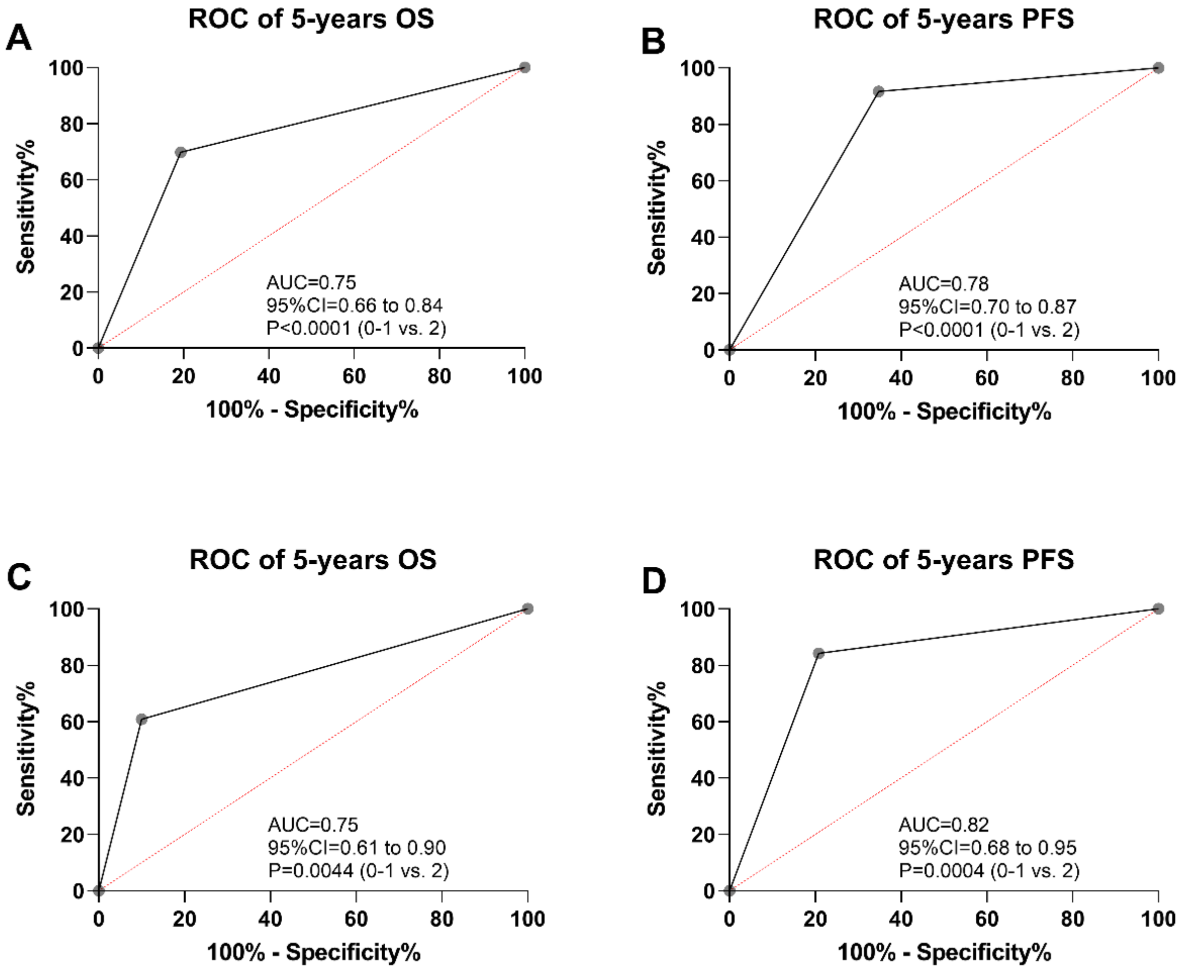

Supplementary Figure 4. ROC curve of MSKCC. (A) ROC curve of OS in the discovery cohort. (B) ROC curve of PFS in the discovery cohort. (C) ROC curve of OS in the validation cohort. (D) ROC curve of PFS in the validation cohort.

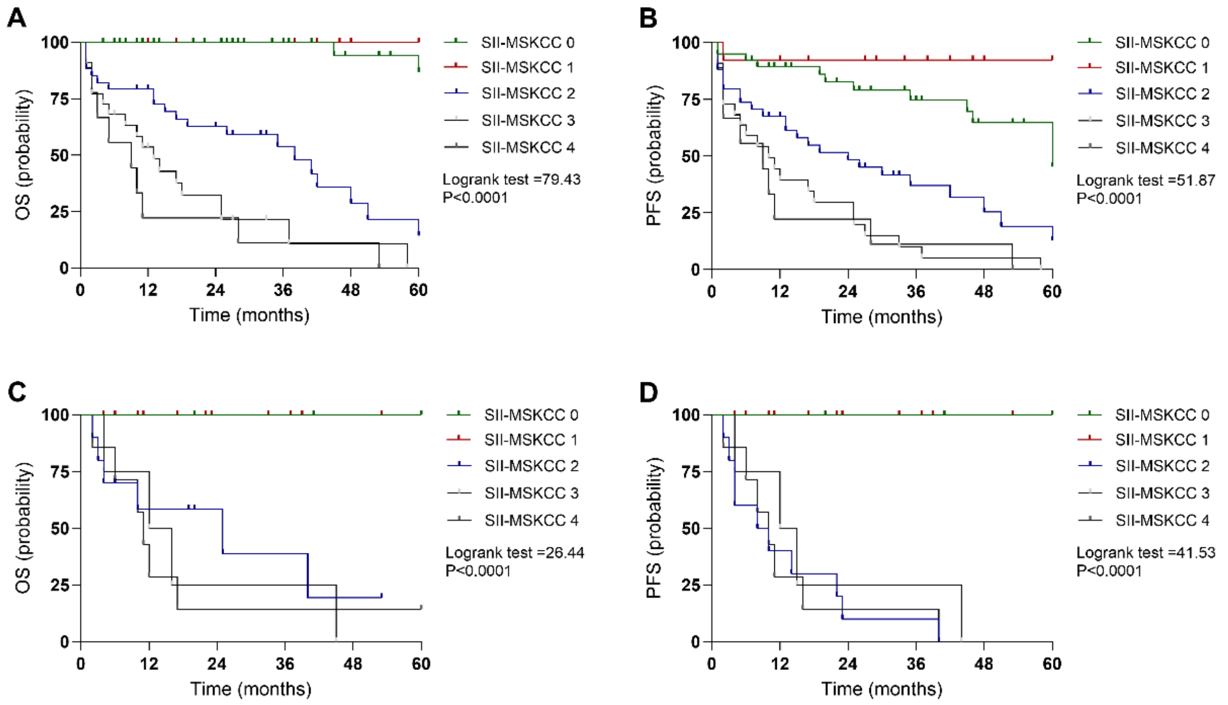

Supplementary Figure 5. Kaplan-Meier estimates of overall survival (OS) and progression-free survival (PFS) according to SII-MSKCC scores. (A) OS according to SII-MSKCC (0-4) five subgroups in discovery cohort. (B) PFS according to SII-MSKCC (0-4) five subgroups in discovery cohort. (C) OS according to SII-MSKCC (0-4) five subgroups in validation cohort. (D) PFS according to SII-MSKCC (0-4) five subgroups in validation cohort.

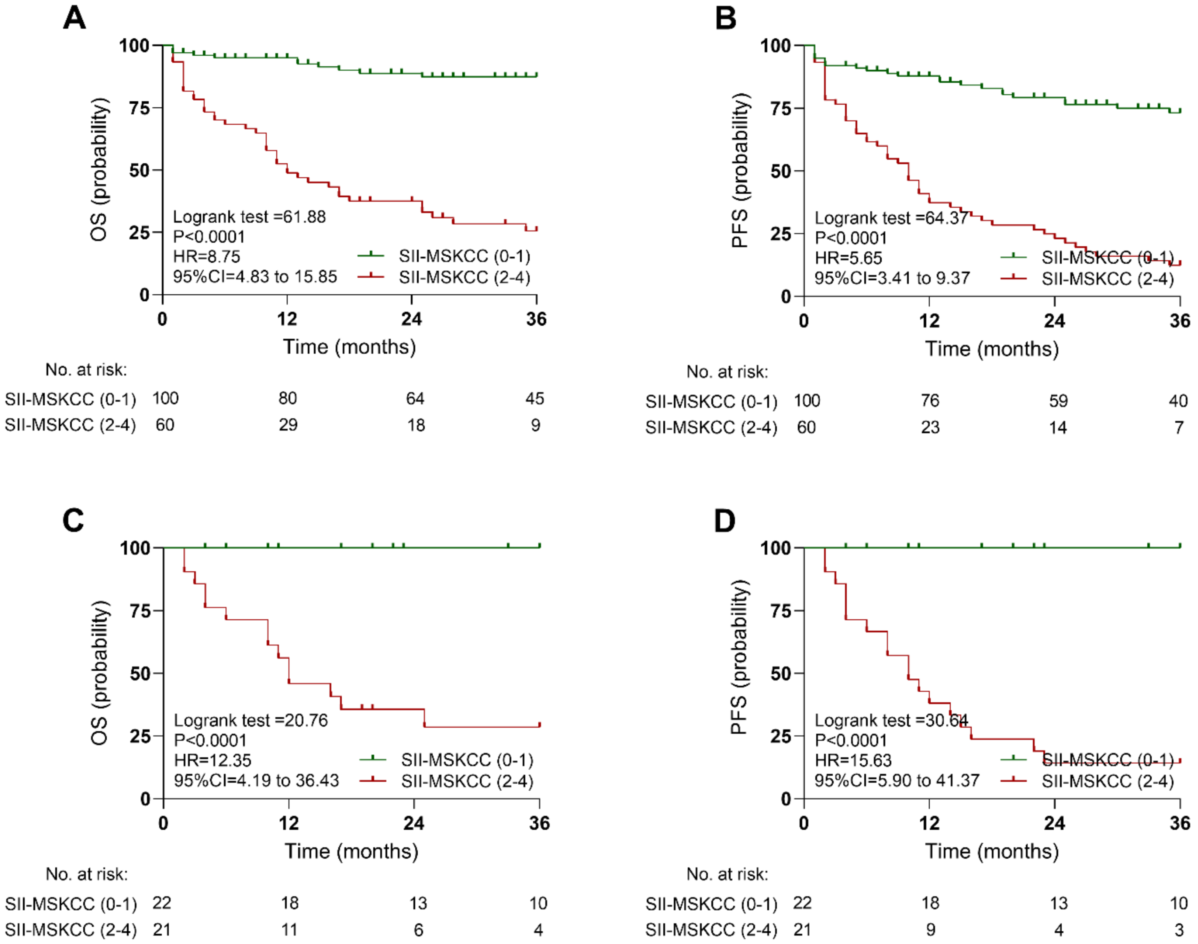

Supplementary Figure 6. Kaplan-Meier estimates 3 years overall survival (OS) and 3 years progression-free survival (PFS) according to SII-MSKCC scores. (A) OS according to SII-MSKCC model scores (0-1 *vs.* 2-4). (B) PFS according to SII-MSKCC scores (0-1 *vs.* 2-4). (C) OS according to SII-MSKCC model scores (0-1 *vs.* 2-4) in the validation cohort. (D) PFS according to SII-MSKCC scores (0-1 *vs.* 2-4) in the validation cohort.

Supplementary Table 1. Demographic and clinical characteristics between discovery cohort and validation cohort

Variables		Discovery Cohort, N (%)	Validation Cohort, N (%)	P value
Number, n		128	46	
Age, Mean±SD, y		61.76±10.71	60.63±11.30	0.55
Male		73 (57)	25 (54)	0.75
Hypertension		41 (32)	15 (33)	0.94
Diabetes mellitus		11 (9)	4 (9)	0.98
BMI, Mean±SD, Kg/m^2		22.86±3.10	23.79±3.16	0.10
IELSG score				
	Missing	30 (23)	12 (26)	
	0-3	82 (64)	30 (65)	0.52
	4-5	16 (13)	4 (9)	
MSKCC score				
	Missing	11 (9)	3 (7)	
	Age≦50 or Age>50+KPS≧70	69 (54)	23 (50)	0.75
	Age>50+KPS<70	48 (38)	20 (43)	
ECOG performance				
	Missing	3 (2)	6 (13)	
	0-2	79 (62)	26 (57)	0.84
	3-5	46 (36)	14 (30)	
IPI score				
	Missing	7 (5)	7 (15)	
	0-2	89 (70)	33 (72)	0.16
	3-5	32 (25)	6 (13)	
LDH				
	Missing	7 (5)	0 (0)	0.77
	Normal	91 (71)	35 (76)	
	Decreased	10 (8)	5 (11)	
	Elevated	20 (16)	6 (13)	
Cell of origin				
	Germinal	27 (21)	16 (35)	0.065
	Non-germinal center	101 (79)	30 (65)	
Treatment				
	MTX	32 (25)	12 (26)	0.88
	MTX-based regimen	96 (75)	34 (74)	
Death				
	Yes	57 (45)	16 (35)	0.25
	No	71 (55)	30 (65)	
Progression				
	Yes	77 (60)	23 (50)	0.23
	No	51 (40)	23 (50)	

BMI: body mass index; LDH: lactic dehydrogenase. Independent student's t-test, and χ^2 tests were used when appropriate.

Supplementary Table 2. The results of immunohistochemistry staining

Center	CD20	CD79a	BCL-2	Bcl-6	MUM-1	PAX-5	Ki67	CyclinD+	CD10
Cancer Center	+		+	+	+	+	80	-	+
Cancer Center	+		+	+	+	+	85		+
Cancer Center	+		+	+	-/+	+	85	-	+
Cancer Center	+		+	+	+		90		+
Cancer Center	+	+	+	+	+		85		+
Cancer Center	+		-	+		+	90		+
Cancer Center	+	+	-	+	+	+	95		+
Cancer Center	+		+	+	+		85	-	+
Cancer Center	+		-	+	+	+	85	-	+
Cancer Center	+	+	+	+	+		85		-
Cancer Center	+		+	+	+	+	85		-
Cancer Center	+		+	+	+		80	-	-
Cancer Center	+	+	+	+	+		85		-
Cancer Center	+		+	+	+		60		-
Cancer Center	+		+	+	+		85	-	-
Cancer Center	+		+	+	+		90	-	-
Cancer Center	+		+	+	+	+	90		-
Cancer Center	+		+	+	+	+	90		-
Cancer Center	+	+	+	+	+		85		-
Cancer Center	+		+	+	+	+	90	-	-
Cancer Center	+		+	+	+		80	-	-
Cancer Center	+		+	+	+		80	-	-
Cancer Center	+	+	+	+	+		85		-
Cancer Center	+		+	+	+	+	70		+
Cancer Center	+		+	+	+	+	90	-	+
Cancer Center	+		+	+	+	+	70		+
Cancer Center	+		+	+/-	+	+	80		+
Cancer Center	+		+	+	+		75		-
Cancer Center	+		+	+	+	+	70	-	-
Cancer Center	+		+	+	+	+	80	-	-
Cancer Center	+	+	+	+	+		85		-
Cancer Center	+	+	+	+	+		85		-
Cancer Center	+		+	+	+		80		-
Cancer Center	+		-	+	+	+	80		-
Cancer Center	+		+	+	+		80	-	-
Cancer Center	+		+	+	+	+	80		-
Cancer Center	+		+	+	+		85		-
Cancer Center	+		+	+	+		60		-
Cancer Center	+		+	+	+	+	85	-	-
Cancer Center	+		+	+	+		90	-	-
Cancer Center	+		-	+	+	+	90	-	+

Cancer Center	+		+	+	+		80		+
Cancer Center	+		+	+	+		80		-
Cancer Center	+		+	+	+		60		-
Cancer Center	+	+	+	+	+		90		-
Cancer Center	+		+	+/-	+		85	-	-
HuaShan Center	+	+		+	+	+	80		+
HuaShan Center	+		-	+	-			-	+
HuaShan Center	+	+	+	+	-	+	80		-
HuaShan Center	+		+	+	+		70		+
HuaShan Center	+	+	+	-	+	+	70		-
HuaShan Center	+	+	+	+	+	+	80		-
HuaShan Center	+	+	-	-	+		60		-
HuaShan Center	+	+	+	+	+		80		-
HuaShan Center	+	+	+	+	+		70	-	-
HuaShan Center	+	+	-	+	+		90		-
HuaShan Center	+	+	+	-	+	+	80		-
HuaShan Center	+	+	-	+	+		70	-	-
HuaShan Center	+	+	+	+	+		70	-	-
HuaShan Center	+	+	-	+	+	+	50		-
HuaShan Center	+	+	+	+	+	+	90		-
HuaShan Center	+	+	+	+	+		50	-	-
HuaShan Center	+	+	+	-	+		90		-
HuaShan Center	+	+	+	-	+		60		+
HuaShan Center	+	+	+	+	+		65		+
HuaShan Center	+	+	+	-	+		60		+
HuaShan Center	+	+	+	+	-	+	70		+
HuaShan Center	+	+		+	+		75		+
HuaShan Center	+	+	-	+	-				-
HuaShan Center	+	+	-	+	-		90	+	-
HuaShan Center	+	+	+	+	+		60	-	-
HuaShan Center	+	+	-	+	+		60		-
HuaShan Center	+	+	-	+	+		70		-
HuaShan Center	+	+	-	+	+	+	50		-
HuaShan Center	+	+	+	+	+	-	80	+	-
HuaShan Center	+		-	+	+	+	90		-
HuaShan Center	+	+	+	+	+	+	90		-
HuaShan Center	+		+	+	+		80		-
HuaShan Center	+			+	+				-
HuaShan Center	+			+	+			-	-
HuaShan Center	+				+			-	-
HuaShan Center	+	+	+	-	+	+	60		-
HuaShan Center	+		+	+	+	+	90	-	-
HuaShan Center	+	+	+	+	+	+	90		-

HuaShan Center	+	+	-	+	+	+	60		-
HuaShan Center	+		-	+	+	+	60		-
HuaShan Center	+	+	-	+	+	+	80		-
HuaShan Center	+	+	+	-	+	+	70	-	-
HuaShan Center	+	+	+	+	+		80		-
HuaShan Center	+	+		+	+		70		-
HuaShan Center	+	+	+	+	+		80		-
HuaShan Center	+	+	+	+	+	+	100	+	-
HuaShan Center	+	+	-	+	-	+	85	-	-
HuaShan Center	+	+	+	+	-	+	70		-
HuaShan Center	+	+	+	+	+		80		-
HuaShan Center	+	+	+	+	+		80		-
HuaShan Center	+	+	-	+	+		90		-
HuaShan Center	+	+	+	+	+	+	80		-
HuaShan Center	+	+	-	+	+		80		-
HuaShan Center	+	+	+	+	+		80	-	-
HuaShan Center	+	+	+	+	+	+	90		-
HuaShan Center	+			+	+			-	-
HuaShan Center	+	+	+	+	+		80		-
HuaShan Center	+			+	+		70		-
HuaShan Center	+	+	-	+	+		60		-
HuaShan Center	+		-	+	+	+	70	-	-
RenJi Center	+	+			+		90		+
RenJi Center	+	+	+	+			80		+
RenJi Center	+	+	+	+	+		90		-
RenJi Center	+	+	+	+	+				-
RenJi Center	+	+	-	-	+	+	70		-
RenJi Center	+	+					70		-
RenJi Center	+		+				40		-
RenJi Center	+	+	+	+	+		85	-	-
RenJi Center	+	+	+	+	+		90		-
RenJi Center	+		+	+	+		70	-	-
RenJi Center	+	+		-	+				-
RenJi Center	+		+	+	+	+	80		-
RenJi Center	+		+	+	+		70		-
RenJi Center	+			-	+		90		-
RenJi Center	+	+	-	-	-		70	-	-
RenJi Center	+	+	+	+		+	80		-
RenJi Center	+		+	+	-		80	-	-
RenJi Center	+		-	+	+		90		+
RenJi Center	+	+		-	+		50		+
RenJi Center	+	+	+	+	+		90	-	+
RenJi Center	+	+	-	+	-		90		-

RenJi Center	+	+	+	+	+		50		-
RenJi Center	+	+					80		-
RenJi Center	+						90	-	-
RenJi Center	+		+	+	+		95		-
RenJi Center	+						80		-
RenJi Center	+	+	+	-	+		80		-
RenJi Center	+	+		-	+		70	-	-
RenJi Center	+	+		-	-		95		-
RenJi Center	+		+	+	+		90	-	-
RenJi Center	+	+	-	+	+		90		-
RenJi Center	+		-	+	+		90		-
RenJi Center	+	+		-	-				-
RenJi Center	+		+	+	+		80		-
RenJi Center	+	+	+						-
RenJi Center	+	+	-	+	+		90		-
RenJi Center	+	+		-	+	+	50	-	-
RenJi Center	+		+				80		-
RenJi Center	+	+	+	+	+		70	-	-
RenJi Center	+	+	+	+	+	+	80		-
RenJi Center	+	+	+	+	+		60		+
RenJi Center	+	+	+	+			90		+
RenJi Center	+		+	+	+		80		+
RenJi Center	+		+	+	-		70		+
RenJi Center	+	+	+	+	-			-	-
RenJi Center	+		+	+	+		70		+
RenJi Center	+		-	+	+		80	-	+
RenJi Center	+	+	+	+	+		80		-
RenJi Center	+	+	+	-			80	-	-
RenJi Center	+	+	+	+	+		90	-	-
RenJi Center	+	+	+	+	+	+	90		-
RenJi Center	+	+	+	+	+		85	-	-
RenJi Center	+	+	+	+	+		90	-	-
RenJi Center	+	+		-	-		70		-
RenJi Center	+	+					90		-
RenJi Center	+		+	+			90		-
RenJi Center	+	+	-	+			70		-
RenJi Center	+	+	-				60	-	-
RenJi Center	+		-	+	+	+	90	-	-
RenJi Center	+			+	+		90		-
RenJi Center	+		+	+	+	+	70		-
RenJi Center	+						80		-
RenJi Center	+		+	+	+		90		-
RenJi Center	+	-	+	+			90		-

RenJi Center	+	+	+	+	+				-
RenJi Center	+	+	+	+	+		70		-
RenJi Center	+		+	+	+		80		-
RenJi Center	+					+	60		-

Supplementary Table 3. Patient characteristics and outcome in discovery cohort by SII groups

Variables	890>SII>450, N (%)	SII>890, N (%)	SII<450, N (%)	P value
Number, n	53	42	33	
Median age (IQR), y	59 (53-69)	64 (57-69)	65 (59-70)	0.47
≤60	29 (49)	16 (40)	10 (40)	
>60	24 (51)	26 (60)	23 (60)	
Male	34 (64)	20 (52)	19 (52)	0.27
Hypertension	19 (36)	13 (29)	9 (29)	0.70
Diabetes mellitus	5 (9)	2 (8)	4 (8)	0.48
Median BMI (IQR), Kg/m^2	22.49 (20.97-24.85)	23.03 (21.09-24.65)	22.15 (19.57-24.96)	0.53
Missing	7 (13)	3 (9)	4 (9)	
<18.5	5 (9)	1 (3)	1 (3)	
18.5-24.0	27 (51)	34 (71)	19 (71)	
>24.0	14 (26)	4 (17)	9 (17)	
IELSG score				
Missing	12 (23)	10 (24)	8 (24)	0.69
0-3	34 (64)	28 (64)	20 (64)	
4-5	7 (13)	4 (12)	5 (12)	
MSKCC score				
Missing	4 (8)	4 (9)	3 (9)	0.86
Age≦50 or Age>50+KPS≧70	30 (57)	21 (52)	18 (52)	
Age>50+KPS<70	19 (36)	17 (39)	12 (39)	
ECOG performance status				<0.0001
Missing	1 (2)	1 (3)	1 (3)	0.016[a]
0	44 (83)	19 (47)	16 (47)	0.0010[b]
1	8 (15)	22 (51)	16 (51)	0.77[c]
IPI score				<0.0001
Missing	3 (6)	2 (5)	2 (5)	<0.0001[a]
0-2	48 (91)	21 (55)	20 (55)	<0.0001[b]
3-5	2 (4)	19 (40)	11 (40)	0.31[c]
LDH				
Missing	2 (4)	3 (7)	2 (7)	0.45
Normal	40 (75)	30 (68)	21 (68)	
Decreased	5 (9)	2 (7)	3 (7)	
Elevated	6 (11)	7 (19)	7 (19)	
Cell of origin				0.88
Missing	0	0	0	
Germinal	12 (23)	9 (20)	6 (20)	
Non-germinal center	41 (77)	33 (80)	27 (80)	
Treatment				0.80
MTX	12 (23)	12 (29)	8 (24)	
MTX-based regimen	41 (77)	30 (71)	25 (76)	
Death				<0.0001
Missing	0	0	0	<0.0001[a]

	Yes	12 (23)	27 (60)	18 (60)	0.0030[b]
	No	41 (77)	15 (40)	15 (40)	0.39[c]
Progression					0.0080
Missing		0	0	0	0.0020[a]
	Yes	24 (45)	32 (71)	21 (71)	0.097[b]
	No	29 (55)	10 (29)	12 (29)	0.24[c]

a: 890>SII>450 vs. SII>890; b: 890>SII>450 vs. SII<450; c: SII>890 vs. SII<450. One way ANOVA, and χ^2 tests were used when appropriate.

Supplementary Table 4. Univariable analysis and multivariable analysis for progression-free survival and overall survival in discovery cohort

	OS		PFS	
	HR (95%CI)	P value	HR (95%CI)	P value
Univariate analysis				
Model 1				
890>SII>450	1		1	
SII<450	3.23 (1.53-6.81)	0.0020	1.82 (1.02-3.23)	0.044
SII>890	4.62 (2.30-9.28)	<0.0001	2.54 (1.48-4.37)	0.0010
Model 2				
890>SII>450	1		1	
SII<450 or SII>890	3.95 (2.07-3.95)	<0.0001	2.13 (1.31-3.45)	0.0022
Model 3				
SII-MSKCC (0-1)	1		1	
SII-MSKCC (2-4)	7.95 (4.17-15.16)	<0.0001	4.89 (2.90-8.15)	<0.0001
Multivariate analysis				
Model 1*				
890>SII>450	1		1	
SII<450	3.11 (1.42-6.82)	0.0050	1.74 (0.96-3.16)	0.069
SII>890	4.54 (2.22-9.28)	<0.0001	2.23 (1.26-3.94)	0.0060
Model 2*				
890>SII>450	1		1	
SII<450 or SII>890	3.80 (1.55-9.31)	0.0041	3.43 (1.56-7.55)	0.0020
Model 3**				
SII-MSKCC (0-1)	1		1	
SII-MSKCC (2-4)	6.44 (2.90-14.31)	<0.0001	3.24 (1.75-5.98)	<0.0001

* Adjusted for sex (male=1, female=2), BMI, MSKCC, diabetes (yes = 1, no = 0), and hypertension (yes = 1, no = 0). ** Adjusted for age, sex (male=1, female=2), BMI, diabetes (yes = 1, no = 0), and hypertension (yes = 1, no = 0). A total of 112 patients (death=53, progression=72) without missing values included in this multivariable analysis model.

Supplementary Table 5. Univariable analysis and multivariable analysis for progression-free survival and overall survival in RenJi Cohort

	OS		PFS	
	HR (95%CI)	P value	HR (95%CI)	P value
Univariate analysis				
Model 1				
890>SII>450	1		1	
SII<450	3.82 (1.40-10.41)	0.0090	3.34 (1.35-8.27)	0.0092
SII>890	3.63 (1.41-9.36)	0.0080	3.67 (1.59-8.47)	0.0021
Model 2				
890>SII>450	1		1	
SII<450 or SII>890	3.72 (1.52-9.06)	0.0043	3.48 (1.59-7.61)	0.0026
Multivariate analysis				
Model 1*				
890>SII>450	1		1	
SII<450	3.86 (1.41-10.56)	0.0090	3.38 (1.36-8.40)	0.0094
SII>890	3.62 (1.39-9.43)	0.0080	3.47 (1.49-8.10)	0.0040
Model 2*				
890>SII>450	1		1	
SII<450 or SII>890	4.40 (1.28-15.11)	0.019	3.43 (1.56-7.55)	0.0020

* Adjusted for gender (male=1, female=2), BMI, MSKCC, diabetes (yes = 1, no = 0), and hypertension (yes = 1, no = 0). A total of 55 patients (death=31, progression=38) without missing values included in this multivariable analysis model.

Supplementary Table 6. Univariable analysis and multivariable analysis for progression-free survival and overall survival in HuaShan Cohort

	OS		PFS	
	HR (95%CI)	P value	HR (95%CI)	P value
Univariate analysis				
Model 1				
890>SII>450	1		1	
SII<450	2.85 (0.93-8.81)	0.065	1.05 (0.47-2.35)	0.90
SII>890	7.62 (2.32-25.03)	0.0010	2.43 (1.08-5.47)	0.032
Model 2				
890>SII>450	1		1	
SII<450 or SII>890	4.23 (1.54-11.65)	0.0050	1.41 (0.72-2.77)	0.31
Multivariate analysis				
Model 1*				
890>SII>450	1		1	
SII<450	4.56 (1.16-7.96)	0.030	0.95 (0.41-2.20)	0.91
SII>890	8.94 (3.28-18.00)	<0.0001	3.03 (1.26-7.30)	0.014
Model 2*				
890>SII>450	1		1	
SII<450 or SII>890	4.58 (1.65-12.69)	0.0030	1.47 (0.74-2.93)	0.27

* Adjusted for gender (male=1, female=2), BMI, MSKCC, diabetes (yes = 1, no = 0), and hypertension (yes = 1, no = 0). A total of 57 patients (death=22, progression=34) without missing values included in this multivariable analysis model.

Supplementary method

GAM, using the mgcv (v1.8-31) package of R software, was used to account for the possible non-linear relationship between the pretreatment SII levels and clinical outcomes. The gamm() function from the mgcv package was perform to analysis the generalized additive mixed modelling, to account for the random effect caused by the clustered (nested) structure of the data. The generalized additive modelling smoother fits polynomial functions between auto selected knots and does not prejudge the relationship between two continuous variables when it cannot be specified by explicit functional, parametric forms. A similar approach has been recently applied in clinical research[1,2]. LOESS can capture significant smoothing features because the local regions are treated independently. While GAM imposes additive structure, requiring that cross sections of the fitted surface always have the same shape. The use of both GAM and LOESS together are more suitable for visualizing the relationship between the pretreatment SII levels and the risk of survival outcomes than traditional linear models. The degree of smoothing was set to 2 (quadratic regression). The span was varied until a curve of a suitable appearance was achieved. We used a smoothing span of 0.6 in the final analysis, span between 0.5 and 0.7 produced extremely similar estimates. A similar approach has been recently applied in clinical research[3,4].

Univariate and multivariate Cox proportional hazards regression models were used to evaluate the association between SII levels and SII-MSKCC with clinical outcome. To include all known potential prognostic covariates thought to affect the risk of clinical outcomes, such as age, sex, body mass index (BMI), MSKCC score, IPI scores, ECOG performance, IELSG score, diabetes, and hypertension, were added to the multivariate cox regression analysis. Owing to age, MSKCC score, IPI scores, ECOG performance, and IELSG score were multicollinearity in multivariate cox regression analysis. Further, on this basis we derived an instrumental variable regression model. Therefore, only sex, BMI, MSKCC score, diabetes, and hypertension variables were included in the multivariate cox regression analysis to ensure the accuracy of the results. Univariate and multivariate Cox proportional hazards regression models were also used to evaluate the association between SII-MSKCC scores and clinical outcome. Age, sex, BMI, diabetes, and hypertension variables were included in the multivariate cox regression analysis. The primary endpoint was evaluated using multivariate Cox proportional hazards regression analysis. The Cox proportional hazards model was used to estimate hazard ratios (HRs) and 95% confidence intervals (CIs) for the survival outcomes. P-values less than 5% were considered statistically significant.

Reference

1. Wand H, Dassaye R, Reddy T, Yssel J, Ramjee G. Geographical-level contributions of risk factors for HIV infections using generalized additive models: results from a cohort of South African women. *AIDS Care*. 2019;31(6):714–722.
2. Galetti V, Stoffel NU, Sieber C, et al. Threshold ferritin and hepcidin concentrations indicating early iron deficiency in young women based on upregulation of iron absorption. *EClinicalMedicine*. 2021;39:101052.
3. Ahern J, Margerison-Zilko C, Hubbard A, Galea S. Alcohol outlets and binge drinking in urban neighborhoods: the implications of nonlinearity for

intervention and policy. *Am J Public Health*. 2013;103(4):e81-87.

4. Prata D, Rodrigues W, De Souza Bermejo PH, et al. The relationship between (sub)tropical climates and the incidence of COVID-19. *PeerJ*. 2021;9:e10655.
